# The Usefulness of the Delta Neutrophil Index for Predicting Superimposed Pneumonia in Patients with Acute Decompensated Heart Failure in the Emergency Department

**DOI:** 10.1371/journal.pone.0163461

**Published:** 2016-09-28

**Authors:** Yong Sung Cha, Kang Hyun Lee, Jong Wook Lee, Woocheol Kwon, Seok Jeong Lee, Kyung Sik Kang, Hyung Il Kim, Oh Hyun Kim, Kyoung-Chul Cha, Hyun Kim, Sung Oh Hwang

**Affiliations:** 1 Department of Emergency Medicine, Yonsei University Wonju College of Medicine, Wonju, Republic of Korea; 2 Department of Laboratory Medicine, Jincheon Sungmo Hospital, Jincheon, Republic of Korea; 3 Department of Radiology, Yonsei University Wonju College of Medicine, Wonju, Republic of Korea; 4 Department of Internal Medicine, Yonsei University Wonju College of Medicine, Wonju, Republic of Korea; Kurume University School of Medicine, JAPAN

## Abstract

**Background:**

Although respiratory infections, such as pneumonia, have long been recognized as precipitators of exacerbation in patients with acute decompensated heart failure (ADHF), identifying signs of concomitant pneumonia in ADHF is a clinical diagnostic challenge. We evaluated the predictive value of the delta neutrophil index (DNI), a new indicator for immature granulocytes, for diagnosing superimposed pneumonia in patients presenting with ADHF in the emergency department (ED).

**Methods:**

This was a retrospective and observational study of consecutive patients (>18 years old) diagnosed with an ADHF in the ED over a 7-month period. Patients were categorized into either the ADHF group or the ADHF with pneumonia group. DNI, serum white blood cell (WBC), C-reactive protein (CRP), and β-natriuretic peptide (BNP) were measured upon ED arrival.

**Results:**

The ADHF with pneumonia group included 30 patients (20.4%). Median initial DNI, WBC, and CRP were significantly higher in the ADHF with pneumonia group [0% vs. 1.8%, p<0.001, 8,200 cells/mL vs. 10,470 cells/mL, p<0.001, and 0.56 mg/dL vs. 6.10 mg/dL, p<0.001]. Multiple logistic regression analyses showed that only initial DNI significantly predicted the presence of superimposed pneumonia in patients with ADHF. In the receiver operating characteristic curves for initial DNI, WBC, and CRP for differentiating superimposed pneumonia in ADHF patients, the area under curve (AUC) of DNI (0.916 [95% confidence interval 0.859–0.955]) was good. AUC of DNI was significantly higher than AUC of CRP and WBC [0.828 and 0.715] (DNI vs. CRP, p = 0.047 and DNI vs. WBC, p<0.001).

**Conclusions:**

Initial DNI, which was measured upon ED arrival, was significantly higher in the ADHF with pneumonia group than in the ADHF group. The initial DNI’s ability of prediction for ADHF with superimposed pneumonia in the ED was good and it was better than those of serum WBC and CRP. Therefore, DNI may serve as a convenient and useful marker for early diagnosis of superimposed pneumonia in patients with ADHF in the ED.

## Introduction

Acute decompensated heart failure (ADHF) is a common and potentially fatal cause of acute respiratory distress. Respiratory infections have long been recognized as precipitators of exacerbation and concomitant pneumonia has been reported in 7–10% of ADHF patients [1–3]. Detecting patients who present with an ADHF and signs of superimposed pneumonia is often difficult because of the non-specific nature of the physical examination or chest x-ray abnormalities in the setting of cardiogenic pulmonary edema. Unfortunately, misdiagnosis may result in delayed antibiotic therapy and may potentially increase morbidity and mortality [4,5]. Conversely, over-treating this unique population with antibiotics is associated with adverse reactions, high cost and the emergence of bacterial resistance [6].

Infectious conditions increase the levels of immature granulocytes. However, this measurement is difficult to obtain in clinical practice because manual counting is not accurate [7–10]. Nahm et al. proved that the delta neutrophil index (DNI), which is the difference in leukocyte subfractions identified by a cytochemical myeloperoxidase reaction and by a nuclear lobularity assay and is determined using a blood cell analyzer (ADVIA 120, Siemens, Inc.), was strongly correlated with manual immature granulocyte counts (r = 0.75, p <0.05) [11–13]. Several investigators examined the ability of DNI to predict early diagnosis, disease severity, and prognosis of patients with sepsis [10,14]. DNI can also be easily used to evaluate inflammation and infection in the ED setting because tests can be performed at the same time as routine complete blood count (CBC). However, no information is available on the clinical usefulness of DNI with respect to the diagnosis of superimposed pneumonia in patients with ADHF.

We hypothesized that DNI might be elevated in patients with superimposed pneumonia in the setting of ADHF. Therefore, we evaluated differences in initial DNI according to the presence of pneumonia in patients with ADHF and the predictive value of DNI for diagnosis of superimposed pneumonia in patients presenting with ADHF at the ED.

## Materials and Methods

### Study setting and population

This was a retrospective and observational study of consecutive patients more than 18 years of age diagnosed with ADHF in the ED over a 7-month period between June 2015 and December 2015. The ED was located in a single urban, tertiary-care hospital (Wonju Severance Christian Hospital, Wonju, Republic of Korea), which has more than 45,000 annual visits and is staffed 24 hours per day by board-certified emergency physicians.

Any patient records in which the word “heart failure” was used as an ED discharge code in computerized hospital records were initially considered for patient selection. A diagnosis of ADHF was made according to a history of previously diagnosed heart failure and an accompanied by specific symptoms, such as dyspnea and fatigue, and signs of fluid retention or new-onset heart failure with symptoms sufficient for the needs of an admission care [15,16]. The selected patients were categorized into the ADHF group or the ADHF with pneumonia group. The ADHF group was defined as patients with ADHF exacerbated by other reasons except for pneumonia. The ADHF with pneumonia group was defined as patients with ADHF exacerbated by concomitant pneumonia. Because radiographic findings in ADHF can range from no specific abnormality to marked cardiomegaly and extensive bilateral interstitial markings [17,18], the radiologic and bacteriological findings were used to define the concomitant pneumonia within the patients. Diagnosis of superimposed pneumonia was made according to the appearance of a new infiltrate not fully explained by ADHF on chest computed tomography (CT), accompanied by a fever of at least 38°C and/or leukocytosis (≥10,000 cells/mL) or leukopenia (≤5,000 cells/mL) and/or positive sputum culture in a patient [19]. The appearance of a new infiltrate on chest CT was confirmed by a specialized radiologist and presence of concomitant pneumonia was retrospectively reviewed by pulmonologist and radiologist. If there were inter-observer disagreements between the interpretations of concomitant pneumonia, both pulmonologist and radiologist reviewed and discussed the cases together for the conclusions.

The study exclusion criteria were: 1) patients with hematologic abnormalities or other concurrent infections and those who received granulocyte colony stimulating factors, glucocorticoids, or other immunosuppressants before study enrollment, which can cause changes in the DNI level, 2) definite evidence of acute ST-elevation myocardial infarction as cause of ADHF, 3) clear alternative diagnosis (foreign body aspiration) as cause of concomitant pneumonia, 4) clinically suspicious for superimposed pneumonia without performing chest CT, 5) ADHF by other concurrent infections except pneumonia, 6) patients transferred from other hospitals due to the effect of other treatments including antibiotics, 7) transfer to another hospital after ED admission, and 8) discharge against medical advice. Since the study was performed retrospectively and observationally, we did not obtained informed consent from the participants and the patient records and/or information were anonymously processed prior to the analysis. Approval of this study was obtained by the institutional review board of Wonju College of Medicine, Yonsei University.

### Data collection

Data were collected by retrospectively reviewing electronic medical records. Data collection was conducted by two emergency physicians who were blinded to the study objectives and hypothesis. The categorization of the patient’s group, which was done by pulmonologist and radiologist, was blinded to the abstractors. The abstractors were trained before a data collection to reduce the possible bias from the data collection. We used explicit case report forms and the chart abstractors and study coordinators were met periodically to resolve any disputes and to review coding rules. The study coordinators monitored the performance of abstractors. We collected demographic data (including age and sex), information regarding preexisting comorbidities, associated symptoms, vital sign (including systolic blood pressure [SBP], diastolic blood pressure, pulse rate, and respiratory rate, body temperature [BT]), and prognosis data (including total admission duration and in-hospital mortality). As laboratory data, DNI, serum white blood cell (WBC) and C-reactive protein (CRP), which are commonly used marker for predicting inflammation and infection in the ED, and β-natriuretic peptide (BNP) were measured upon ED arrival.

The ADVIA 120/2120 automatic cell analyzer (Siemens, Tarrytown, NY, USA) was used for determining DNI. It is specific to the technology found in the ADVIA unit manufactured by Siemens, which is a flow cytometry-based hematologic analyzer that uses two independent WBC analysis methods using a myeloperoxidase (MPO) channel and a lobularity/nuclear density channel. DNI was calculated in leukocyte differentials using the following formula: DNI = (the leukocyte subfraction assayed in the MPO channel by cytochemical reaction)–(the leukocyte subfraction counted in the nuclear lobularity channel by the reflected light beam) [11–13].

The primary outcome of this study was the investigation of differences in initial DNI between the ADHF group and the ADHF with pneumonia group, and the predictive ability of the initial DNI measured upon ED arrival for differentiating superimposed pneumonia in patients with ADHF.

### Data analysis

Categorical variables are presented as frequencies and percentages, and continuous variables are presented as means and standard deviations (SD) or as medians and interquartile ranges (IQR). Normality was assessed using the Shapiro-Wilk test. The chi-square test or Fisher’s exact test was used to compare categorical variables, while the two-sample t-test or the Mann-Whitney U test were used to compare continuous variables. The area under curve (AUC) for the predictive ability for the presence of concomitant pneumonia was determined using receiver operating characteristic (ROC) curves. In addition, comparison of AUCs was used to compare the predictive ability of each method for the presence of superimposed pneumonia in ADHF. P-values of <0.05 were considered statistically significant, and the analysis was performed using SPSS Ver. 20 (IBM, Armonk, NY, USA) and SAS 9.2 Ver. (SAS Institute Inc., Cary, NC, USA).

## Results

### Characteristics of study subjects

A total of 218 consecutive patients, aged over 18 years old, were identified with ADHF during the study period. The following patients were excluded: those with hematologic abnormalities including leukemia or myelodysplastic syndrome (10 patients), definite evidence of acute ST-elevation myocardial infarction (11 patients), a clear alternative diagnosis (foreign body aspiration) (9 patients), clinically suspicious for superimposed pneumonia without performing chest CT (2 patients), another concurrent infection like urinary tract or gastrointestinal infection (10 patients), patients transferred from another hospital due to other treatment effects including antibiotics (17 patients), patients transferred to another hospital after ED admission (5 patients), patients discharged against medical advice (5 patients), and those with insufficient data (2 patients). Ultimately, we included 147 patients of the 218 patients with ADHF.

The baseline characteristics of the 147 study subjects are shown in Table 1. Fifty-eight patients were male (39.5%) and the overall median age was 79 years. Common medical past histories were hypertension (63.9%) and coronary artery disease (27.9%). Common initial symptoms at ED presentation were dyspnea (90.5%), chest discomfort (44.2%), and cough (38.8%). Median SBP and BT were 135 mmHg and 36.5°C, respectively. Five patients (5.9%) died in the hospital despite treatment.

**Table 1:** Baseline characteristics and laboratory findings of patients with ADHF.

Characteristics	Total (n = 147)	ADHF group	ADHF with pneumonia group	p-value
		(n = 117; 79.6%)	(n = 30; 20.4%)	
Age (years)	79.0 (74.0–83.0)*	79.0 (74.0–84.0)*	78.0 (73.3–80.3) *	0.297
Male	58 (39.5%)	38 (32.5%)	20 (66.7%)	**0.001**
Past history				
DM	38 (25.9%)	33 (28.2%)	5 (16.7%)	0.198
HTN	94 (63.9%)	77 (65.8%)	17 (56.7%)	0.352
CAD	41 (27.9%)	34 (29.1%)	7 (23.3%)	0.533
Hyperlipidemia	20 (13.6%)	17 (14.5%)	3 (10.0%)	0.766
COPD	13 (8.8%)	5 (4.3%)	8 (26.7%)	**0.001**
Asthma	12 (8.2%)	7 (6.0%)	5 (16.7%)	0.069
Symptoms				
Dyspnea	133 (90.5%)	107 (91.5%)	26 (86.7%)	0.485
Cough	57 (38.8%)	40 (34.2%)	17 (56.7%)	**0.024**
Sputum	46 (31.3%)	31 (26.5%)	15 (50.0%)	**0.013**
Fever	14 (9.5%)	6 (5.1%)	8 (26.7%)	**0.002**
Chilling	17 (11.6%)	11 (9.4%)	6 (20.0%)	0.117
Chest discomfort	65 (44.2%)	61 (52.1%)	4 (13.3%)	**<0.001**
Edema	28 (19.0%)	23 (19.7%)	5 (16.7%)	0.710
General weakness	51 (34.7%)	42 (35.9%)	9 (30.0%)	0.545
Vital sign				
SBP (mmHg)	135 (117–152)*	136 (118–153)*	131 (106–150)*	0.159
DBP (mmHg)	75 (63–87)*	74 (64–90)*	76 (60–85)*	0.420
PR (rates/min)	100 (80–117)*	99 (78–116)*	109 (85–130)*	0.132
RR (rates/min)	20 (18–22)*	20 (20–22)*	20 (18–24)*	0.960
BT (°C)	36.5 (36.1–36.8)*	36.4 (36.1–36.8)*	36.7 (36.3–37.4)*	**0.022**
Laboratory tests				
DNI (%)	0 (0–0.2)*	0 (0–0)*	1.8 (0.8–3.8)*	**<0.001**
WBC (cells/mL)	8,570 (6,590–10,710)*	8,200 (6,000–10,330)*	10,470 (8,078–13,873)*	**<0.001**
CRP (mg/dL)	0.92 (0–3.39)*	0.56 (0–1.71)*	6.10 (1.60–16.93)*	**<0.001**
BNP (pg/mL)	1,078 (556–1,768)*	1,193 (652–1,855)*	727 (357–1,286)*	**0.017**
Total admission days	6.0 (4.0–10.0)*	5.0 (3.0–9.0)*	9.5 (5.8–13.0)*	**0.001**
In-hospital mortality	5 (3.4%)	2 (1.7%)	3 (10.0%)	0.058

ADHF, acute decompensated heart failure; DM, diabetes mellitus; HTN, hypertension; CAD, coronary artery disease; COPD, chronic obstructive lung disease; SBP, systolic blood pressure; DBP, diastolic blood pressure; PR, pulse rate; RR, respiratory rate; BT, body temperature; DNI, delta neutrophil index; WBC, white blood cell; CRP, C-reactive protein; BNP, β-natriuretic peptide

* Median (interquartile range).

The ADHF with pneumonia group included 30 patients (20.4%). The results of the univariate analysis between the ADHF group and the ADHF with pneumonia group are shown in Table 1. Patients in the ADHF group and the ADHF with pneumonia group differed significantly in terms of male (32.5% vs. 66.7%, p = 0.001), chronic obstructive pulmonary disease (COPD) (4.3% vs. 26.7%, p<0.001), and total admission days (5.0 days vs. 9.5 days, p = 0.001) (Table 1).

### Main results

**Fig 1:**
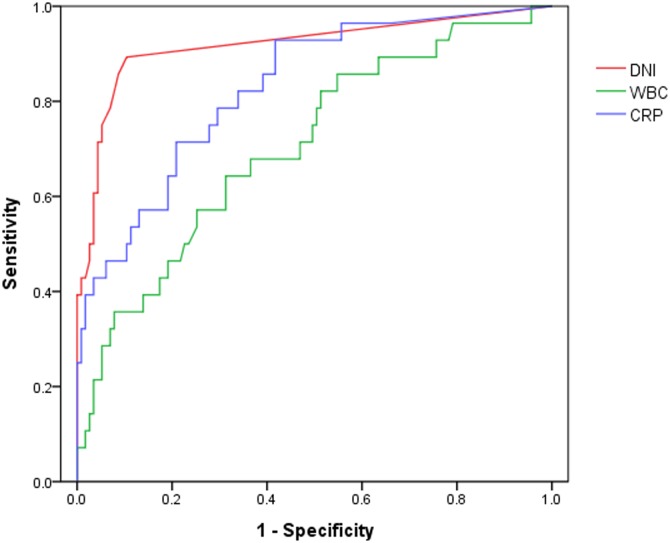
ROC curves of inflammatory markers for predicting ADHF with pneumonia. ROC, receiver operating characteristic; ADHF, acute decompensated heart failure; DNI, delta neutrophil index; WBC, white blood cell; CRP, C-reactive protein

Median initial DNI was significantly higher in the ADHF with pneumonia group than in the ADHF group (1.8% vs. 0%, p<0.001). Also, the median initial serum WBC and CRP were significantly higher in the ADHF with pneumonia group than in the ADHF group (10,470 cells/mL vs. 8,200 cells/mL, p<0.001 and 6.10 mg/dL vs. 0.56 mg/dL, p<0.001). In contrast, serum BNP was significantly higher in the ADHF group than in the ADHF with pneumonia group (1,193 pg/mL vs. 727 pg/mL, p<0.001) (Table 1). Multiple logistic regression analyses showed that only initial DNI significantly predicted the presence of superimposed pneumonia in patients with ADHF in the ED (Table 2). The areas under the ROC curves for initial DNI, CRP, and WBC for differentiating the ADHF group from the ADHF with pneumonia group were 0.916 (95% confidence intervals [CI] 0.859–0.955), 0.828 (95% CI 0.756–0.886), and 0.715 (95% CI 0.635–0.786), respectively. The AUC of initial DNI was significantly higher than the AUC of initial serum WBC and CRP in terms of predicting ADHF with pneumonia (p<0.001 and p = 0.047). (Table 3 and Fig 1).

**Table 2:** Predictor of superimposed pneumonia in patients with ADHF as determined by multivariate logistic regression analysis.

Variables	OR	95% CI	p-value
Male	2.866	0.634–12.952	0.171
COPD	2.153	0.196–23.648	0.530
Cough	0.361	0.056–2.314	0.282
Sputum	1.717	0.297–9.912	0.546
Fever	5.661	0.699–45.834	0.104
Chest discomfort	0.255	0.052–1.241	0.090
DNI (%)	3.247	1.537–6.858	**0.002**
WBC (cells/mL)	1.045	0.854–1.280	0.668
CRP (mg/dL)	1.139	0.973–1.332	0.105
BNP (pg/mL)	1.000	0.999–1.000	0.321

ADHF, acute decompensated heart failure; OR, odds ratio; CI, confidence interval; COPD, chronic obstructive pulmonary disease; DNI, delta neutrophil index; WBC, white blood cell; CRP, C-reactive protein; BNP, β-natriuretic peptide

**Table 3:** Values of the ROC curves for DNI, WBC, and CRP for predicting superimposed pneumonia in patients with ADHF.

Variables	AUC (95% CI)	p-value (vs. WBC)	p-value (vs. CRP)
DNI	0.916 (0.859–0.955)	**<0.001**	**0.047**
CRP	0.828 (0.756–0.886)	**0.028**	-
WBC	0.715 (0.635–0.786)	-	

ROC, receiver operating characteristic; DNI, delta neutrophil index; WBC, white blood cell; CRP, C-reactive protein; ADHF, acute decompensated heart failure; AUC, area under the curve; CI, confidence interval

## Discussion

To the best of our knowledge, this is first report to evaluate the relationship between initial DNI and superimposed pneumonia in ADHF. In this study, initial DNI among patients with ADHF on presentation at the ED was significantly higher in patients with pneumonia compared to those without pneumonia. Superimposed pneumonia can cause increased levels of immature granulocytes due to infection and inflammation. Therefore, DNI, which correlates well with immature granulocyte levels, may be increased in the ADHF with pneumonia group [7,8,11]. The present study shows that initial DNI is a good predictor of superimposed pneumonia in patients with ADHF in the ED, and the predictive ability of the initial DNI for the ADHF with pneumonia group was good. Also, the prediction rate of the initial DNI was significantly higher than that of initial serum WBC and CRP, which are commonly used markers for predicting inflammation and infection. Based on our data, DNI showed higher accuracy than WBC and CRP for detection of superimposed pneumonia with ADHF in the ED. The process of granular leukocyte differentiation in an infectious condition starts from immature granulocyte formation; therefore, the change in DNI may have preceded the change in absolute number of WBC or neutrophil [14]. DNI analysis has another important merit in that it does not require any additional time or cost in the clinical setting, because it is performed routinely along with leukocyte differential counts, and the results can be obtained at the same time as WBC counts and neutrophil fractions in CBC testing. Therefore, we suggest that initial DNI might be used as an additional parameter to differentiate ADHF with superimposed pneumonia from ADHF in the ED.

Serum CRP is a sensitive marker of pneumonia, and its measurement is considered common practice. Increased serum CRP on admission has been demonstrated to be a relatively more sensitive marker in pneumonia patients than any clinical symptom or sign [20–23]. Joffe et al. reported that the mean serum CRP level on admission was 1.35±1.35 mg/dL for ADHF patients and 12.7±8.4 mg/dL for ADHF with superimposed pneumonia patients (p<0.001). Although CRP elevation has also been noted in HF patients, the prediction rate of the differentiating ability between ADHF and ADHF with superimposed pneumonia (AUC 0.918) was good compared with common practice clinical and radiological criteria [24]. However, Simon et al. reported that serum CRP has low sensitivity and specificity for bacterial infection [25]. In the present study, the prediction rate of initial serum CRP was lower than that of DNI and was not a predictor of superimposed pneumonia with ADHF, although there was a significant difference in serum CRP between the ADHF group and the pneumonia with ADHF group.

BNP and N-terminal pro-BNP (NT-proBNP) assays can supplement clinical judgment when the cause of a patient's dyspnea is uncertain, particularly among patients with an intermediate probability of HF [26,27]. In this study, BNP was significantly higher in the ADHF group than in the superimposed pneumonia group. However, Yang et al. reported that NT-proBNP was significantly higher in the superimposed pneumonia group than in the ADHF group (3,147 pg/mL vs. 7,039 pg/mL, p<0.0001) [28]. We thought that change from the baseline value of BNP or NT-proBNP was more important than the absolute value of BNP or NT-proBNP, because the baseline value may differ according to the disease status of the patient.

In this study, the prevalence of ADHF with pneumonia (20.4%) was higher than in other reports (7–10%) [1–3]. Differences in prevalence may develop depending on the study population and the criteria used to define ADHF and superimposed pneumonia. In this study, the ADHF with pneumonia group had more lung diseases, including COPD; in a study by Joffe et al., there were no differences in terms of lung disease, such as COPD and asthma [24]. We believe these differences may have resulted from the fact that diseases and conditions in the host, such as COPD, may lead to impairment of the pulmonary defense and increased risk of community-acquired pneumonia [29]. In this study, total admission days were significantly higher in the superimposed pneumonia group. We thought that, because the ADHF with pneumonia group had significantly more underlying lung disease, they may have had lower lung function than the ADHF group. Iverson and colleagues have shown a clear link between lung function and outcome in HF patients by demonstrating the prognostic importance of spirometric variables, in addition to known risk factors, including self-reported COPD [30]. Therefore, this may account for the greater total admission days in the ADHF with pneumonia group than in the ADHF group.

This study has some limitations. First, the present study was limited by its retrospective design. During data collection, some data might be missing data. Also, since this study was conducted at the emergency center of a single hospital, the sample size was small. Second, selection bias could have been caused by the excluded patients. Third, we did not classify patients in the ADHF with pneumonia group according to infection severity. Seok et al. reported that the median DNI value was 0.0% in a control (no evidence of infection or inflammation) group, 0.8% in a systemic inflammatory response syndrome group, 3.4% in a sepsis group, and 18.6% in a severe sepsis group. Furthermore, there were significant differences among the groups [10]. Therefore, we believe that median initial DNI value might be different according to infection severity. Fourth, we did not evaluate the time from symptom onset to ED arrival. It is possible that this time gap could affect the values of the inflammatory markers. Fifth, because serial DNI values were not investigated after admission, we did not evaluate the usefulness of changes in DNI. Sixth, we could not investigate baseline BNP values in patients with ADHF. Therefore, we could not evaluate changes from the baseline value of BNP and the usefulness of changes in BNP. Seventh, in Maisel et al., it was reported that procalcitonin may aid in the decision to administer antibiotics therapy to patients presenting with acute heart failure in whom clinical uncertainly existed regarding a superimposed bacterial infection [31]. However, we did not investigate serum procalcitonin in all of the included patients. Therefore, we could not compare the usefulness of DNI with the usefulness of procalcitonin. Although the ADHF with pneumonia group had higher serum procalcitonin values than the ADHF group, there was no significant difference between the ADHF group and the ADHF with pneumonia group. We believe that a well-designed prospective study is needed to expand our current knowledge and overcome the limitations of our current study.

## Conclusions

Initial DNI, which was measured upon ED arrival, was significantly higher in the ADHF with pneumonia group than in the ADHF group, and the predictive ability of initial DNI for ADHF with superimposed pneumonia in the ED was better than those of serum WBC and CRP. Therefore, DNI may serve as a convenient and useful marker for early diagnosis of superimposed pneumonia in patients with ADHF in the ED.

## References

[1] Khand AU, Gemmell I, Rankin AC, Cleland JG. Clinical events leading to the progression of heart failure: insights from a national database of hospital discharges. Eur Heart J. 2001;22: 153–164. 10.1053/euhj.2000.2175 11161917

[2] Hugli O, Braun JE, Kim S, Pelletier AJ, Camargo CA Jr. United States emergency department visits for acute decompensated heart failure, 1992 to 2001. Am J Cardiol. 2005;96: 1537–1542. 10.1016/j.amjcard.2005.07.064 16310436

[3] Tsuyuki RT, McKelvie RS, Arnold JM, Avezum A Jr., Barretto AC, Carvalho AC, et al Acute precipitants of congestive heart failure exacerbations. Arch Intern Med. 2001;161: 2337–2342. 11606149 10.1001/archinte.161.19.2337

[4] Wuerz RC, Meador SA. Effects of prehospital medications on mortality and length of stay in congestive heart failure. Ann Emerg Med. 1992;21: 669–674. 1590605 10.1016/s0196-0644(05)82777-5

[5] Mueller C, Scholer A, Laule-Kilian K, Martina B, Schindler C, Buser P, et al Use of B-type natriuretic peptide in the evaluation and management of acute dyspnea. N Engl J Med. 2004;350: 647–654. 10.1056/NEJMoa031681 14960741

[6] Organization WH. World Health Organization report on infectious diseases 2000—overcoming antibiotic resistance. World Health Organization, Geneva, Switzerland. http://www.who.int/infectious-disease-report/2000/index.html. 2000:

[7] Ansari-Lari MA, Kickler TS, Borowitz MJ. Immature granulocyte measurement using the Sysmex XE-2100. Relationship to infection and sepsis. Am J Clin Pathol. 2003;120: 795–799. 10.1309/lt30-bv9u-jjv9-cfhq 14608908

[8] Cornbleet PJ. Clinical utility of the band count. Clin Lab Med. 2002;22: 101–136. 11933571 10.1016/s0272-2712(03)00069-6

[9] Kim HW, Ku S, Jeong SJ, Jin SJ, Han SH, Choi JY, et al Delta neutrophil index: could it predict mortality in patients with bacteraemia? Scand J Infect Dis. 2012;44: 475–480. 10.3109/00365548.2012.657232 22339622

[10] Seok Y, Choi JR, Kim J, Kim YK, Lee J, Song J, et al Delta neutrophil index: a promising diagnostic and prognostic marker for sepsis. Shock. 2012;37: 242–246. 10.1097/SHK.0b013e3182454acf 22258230

[11] Nahm CH, Choi JW, Lee J. Delta neutrophil index in automated immature granulocyte counts for assessing disease severity of patients with sepsis. Ann Clin Lab Sci. 2008;38: 241–246. 18715852

[12] Kratz A, Maloum K, O'Malley C, Zini G, Rocco V, Zelmanovic D, et al Enumeration of nucleated red blood cells with the ADVIA 2120 Hematology System: an International Multicenter Clinical Trial. Lab Hematol. 2006;12: 63–70. 10.1532/lh96.06010 16751132

[13] Harris N, Jou JM, Devoto G, Lotz J, Pappas J, Wranovics D, et al Performance evaluation of the ADVIA 2120 hematology analyzer: an international multicenter clinical trial. Lab Hematol. 2005;11: 62–70. 10.1532/lh96.04064 15790554

[14] Park BH, Kang YA, Park MS, Jung WJ, Lee SH, Lee SK, et al Delta neutrophil index as an early marker of disease severity in critically ill patients with sepsis. BMC Infect Dis. 2011;11: 299 10.1186/1471-2334-11-299 22040292 PMC3213213

[15] Adams KF Jr., Fonarow GC, Emerman CL, LeJemtel TH, Costanzo MR, Abraham WT, et al Characteristics and outcomes of patients hospitalized for heart failure in the United States: rationale, design, and preliminary observations from the first 100,000 cases in the Acute Decompensated Heart Failure National Registry (ADHERE). Am Heart J. 2005;149: 209–216. 10.1016/j.ahj.2004.08.005 15846257

[16] Tan LB, Williams SG, Tan DK, Cohen-Solal A. So many definitions of heart failure: are they all universally valid? A critical appraisal. Expert Rev Cardiovasc Ther. 2010;8: 217–228. 10.1586/erc.09.187 20136608

[17] Badgett RG, Mulrow CD, Otto PM, Ramírez G. How well can the chest radiograph diagnose left ventricular dysfunction? Journal of general internal medicine. 1996;11: 625–634. 8945695 10.1007/BF02599031

[18] Collins SP, Lindsell CJ, Storrow AB, Abraham WT, Committee ASA, Investigators, et al Prevalence of negative chest radiography results in the emergency department patient with decompensated heart failure. Annals of emergency medicine. 2006;47: 13–18. 10.1016/j.annemergmed.2005.04.003 16387212

[19] Niederman MS, Mandell LA, Anzueto A, Bass JB, Broughton WA, Campbell GD, et al Guidelines for the management of adults with community-acquired pneumonia. Diagnosis, assessment of severity, antimicrobial therapy, and prevention. Am J Respir Crit Care Med. 2001;163: 1730–1754. 10.1164/ajrccm.163.7.at1010 11401897

[20] Smith RP, Lipworth BJ, Cree IA, Spiers EM, Winter JH. C-reactive protein. A clinical marker in community-acquired pneumonia. Chest. 1995;108: 1288–1291. 7587431 10.1378/chest.108.5.1288

[21] Almirall J, Bolibar I, Toran P, Pera G, Boquet X, Balanzo X, et al Contribution of C-reactive protein to the diagnosis and assessment of severity of community-acquired pneumonia. Chest. 2004;125: 1335–1342. 15078743 10.1378/chest.125.4.1335

[22] Hopstaken RM, Muris JW, Knottnerus JA, Kester AD, Rinkens PE, Dinant GJ. Contributions of symptoms, signs, erythrocyte sedimentation rate, and C-reactive protein to a diagnosis of pneumonia in acute lower respiratory tract infection. Br J Gen Pract. 2003;53: 358–364. 12830562 PMC1314594

[23] Castro-Guardiola A, Armengou-Arxe A, Viejo-Rodriguez A, Penarroja-Matutano G, Garcia-Bragado F. Differential diagnosis between community-acquired pneumonia and non-pneumonia diseases of the chest in the emergency ward. Eur J Intern Med. 2000;11: 334–339. 11113658 10.1016/s0953-6205(00)00118-7

[24] Joffe E, Justo D, Mashav N, Swartzon M, Gur H, Berliner S, et al C-reactive protein to distinguish pneumonia from acute decompensated heart failure. Clin Biochem. 2009;42: 1628–1634. 10.1016/j.clinbiochem.2009.08.007 19703436

[25] Simon L, Gauvin F, Amre DK, Saint-Louis P, Lacroix J. Serum procalcitonin and C-reactive protein levels as markers of bacterial infection: a systematic review and meta-analysis. Clinical Infectious Diseases. 2004;39: 206–217. 10.1086/421997 15307030

[26] Lindenfeld J, Albert NM, Boehmer JP, Collins SP, Ezekowitz JA, Givertz MM, et al HFSA 2010 Comprehensive Heart Failure Practice Guideline. J Card Fail. 2010;16: e1–194. 10.1016/j.cardfail.2010.04.004 20610207

[27] Weintraub NL, Collins SP, Pang PS, Levy PD, Anderson AS, Arslanian-Engoren C, et al Acute heart failure syndromes: emergency department presentation, treatment, and disposition: current approaches and future aims: a scientific statement from the American Heart Association. Circulation. 2010;122: 1975–1996. 10.1161/CIR.0b013e3181f9a223 20937981

[28] Yang S, Li L, Cao J, Yu H, Xu H. The differential diagnostic value of serum NT-proBNP in hospitalized patients of heart failure with pneumonia. J Clin Lab Anal. 2015;29: 37–42. 10.1002/jcla.21724 24687945 PMC6807215

[29] Almirall J, Bolibar I, Balanzo X, Gonzalez CA. Risk factors for community-acquired pneumonia in adults: a population-based case-control study. Eur Respir J. 1999;13: 349–355. 10065680 10.1183/09031936.99.13234999

[30] Iversen KK, Kjaergaard J, Akkan D, Kober L, Torp-Pedersen C, Hassager C, et al The prognostic importance of lung function in patients admitted with heart failure. Eur J Heart Fail. 2010;12: 685–691. 10.1093/eurjhf/hfq050 20395261

[31] Maisel A, Neath SX, Landsberg J, Mueller C, Nowak RM, Peacock WF, et al Use of procalcitonin for the diagnosis of pneumonia in patients presenting with a chief complaint of dyspnoea: results from the BACH (Biomarkers in Acute Heart Failure) trial. Eur J Heart Fail. 2012;14: 278–286. 10.1093/eurjhf/hfr177 22302662 PMC3284113

